# Association of Achilles tendon thickness and LDL-cholesterol levels in patients with hypercholesterolemia

**DOI:** 10.1186/s12944-018-0765-x

**Published:** 2018-06-01

**Authors:** Bei Wang, Qiuwang Zhang, Ling Lin, Li-li Pan, Cheng-yu He, Xiang-xin Wan, Zhi-ang Zheng, Zheng-xin Huang, Chao-bao Zou, Ming-chang Fu, Michael J. Kutryk

**Affiliations:** 1 Department of Cardiology, the Third People′s Hospital of Hainan Province, 1154 Jiefang Road, Sanya, 572000 Hainan China; 2 Division of Cardiology, Keenan Research Center for Biomedical Science at the Li Ka Shing Knowledge Institute, St. Michael′s Hospital, University of Toronto, Toronto, Ontario Canada; 3 Department of Radiology, the Third People′s Hospital of Hainan Province, Sanya, Hainan China; 4 Department of Laboratory Medicine, the Third People′s Hospital of Hainan Province, Sanya, Hainan China

**Keywords:** Low density lipoprotein cholesterol, Hypercholesterolemia, Achilles tendon xanthoma, Achilles tendon thickness, Cardiovascular disease, Digital radiography

## Abstract

**Background:**

Achilles tendons are the most common sites of tendon xanthomas that are commonly caused by disturbance of lipid metabolism. Achilles tendon thickening is the early characteristic of Achilles tendon xanthomas. The relationship between Achilles tendon thickness (ATT) and LDL-C levels, and risk factors of ATT in patients with hypercholesterolemia, have thus far been poorly documented.

**Methods:**

A total of 205 individuals, aged 18-75 years, were enrolled from March 2014 to March 2015. According to the LDL-C levels and the “Chinese Guidelines on Prevention and Treatment of Dyslipidemia in Adults”, all subjects were divided into 3 groups: normal group (LDL-C < 3.37 mmol/L, *n* = 51); borderline LDL-C group (3.37 mmol/L ≤ LDL-C ≤ 4.12 mmol/L, *n* = 50); and hypercholesterolemia group (LDL ≥ 4.14 mmol/L, *n* = 104). ATT was measured using a standardized digital radiography method and the results were compared among the 3 groups. The correlation between ATT and serum LDL-C levels was analyzed by Pearson’s correlation, and the risk factors of ATT were determined by the logistic regression model.

**Results:**

ATT in borderline LDL-C group was 8.24 ± 1.73 mm, markedly higher than 6.05 ± 0.28 mm of normal group (*P* < 0.05). ATT in hypercholesterolemia group was 9.42 ± 3.63 mm which was significantly higher than that of normal group (*P* < 0.005) and that of borderline LDL-C group (*P* < 0.05). There was a positive correlation between the serum LDL-C levels and ATT (*r* = 0.346, *P* < 0.001). The serum LDL-C level was a risk factor (OR = 1.871, 95% CI: 1.067-3.280) while the levels of HDL-C (OR = 0.099, 95% CI: 0.017-0.573) and Apo AI (OR = 0.035, 95% CI: 0.003-0.412) were protective factors of ATT.

**Conclusions:**

ATT might serve as a valuable auxiliary diagnostic index for hypercholesterolemia and used for the assessment and management of cardiovascular disease.

## Background

Tendinous xanthomas, consisting mainly of lipids and monocyte-derived foam cells, are commonly caused by lipoprotein metabolism disorders such as familial hypercholesterolemia (FH) [1]. Tendon xanthomas are independently associated with the presence and burden of coronary atherosclerosis [2–5]. The Achilles tendon, the strongest and thickest tendon in the body, is the most common site of tendonous xanthomas [6]. An early sign of Achilles tendon xanthomas is Achilles tendon thickening, which can be quantitatively measured by standardized digital radiography (DR) [7, 8]. It has been shown that Achilles tendon thickness (ATT) is a risk factor of cardiovascular disease (CVD) in patients with FH [9]. However, the relationship between ATT and the degree of hypercholesterolemia particularly that of low density lipoprotein cholesterol (LDL-C), and factors associated with ATT in patients with hypercholesterolemia have thus far been poorly documented.

In this study, we used a standardized digital radiography method to measure ATT in patients with hypercholesterolemia and in subjects with normal and borderline LDL-C, and compared the results. The relationship between ATT and LDL-C levels, and positive and negative predictors for ATT are described.

## Methods

### Study subjects

A total of 205 individuals, aged 18-75 years, were enrolled between March 2014 and March 2015. Based on LDL-C levels, subjects were assigned to one of three tertiles described by the Joint Committee for Developing Chinese guidelines on Prevention and Treatment of Dyslipidemia in Adults [10]: normal LDL-C (LDL-C < 3.37 mmol/L, *n* = 51); borderline LDL-C (3.37 mmol/L ≤ LDL-C ≤ 4.12 mmol/L, *n* = 50); and high LDL-C (LDL ≥ 4.14 mmol/L, *n* = 104). A pilot study of 8 participants in each tertile was first performed to determine sample size, which showed that the ATT mean value in normal group, borderline LDL-C group and high LDL-C group was 5.88 mm, 7.50 mm and 8.89, respectively, with the highest standard deviation of 1.25 seen in the high LDL-C group. Using these preliminary data, the sample size required to detect a significant difference in ATT was calculated with an online program (http://powerandsamplesize.com/Calculators/Compare-k-Means/1-Way-ANOVA-Pairwise), and the results showed that 15 subjects in each group are needed, ensuring that the power of the test is 80% with type I error of 0.05. All patients with hypercholesterolemia (high LDL-C group) were newly diagnosed, and had not received any lipid-lowering therapy prior to participating in this study. None of the subjects in the normal or borderline LDL-C groups were taking lipid-lowering therapy. Patients with conditions that could affect Achilles tendon thickness such as tendinitis, tenosynovitis, bursitis, tuberculum arthriticum, rheumatoid arthritis, Achilles tendon injury or prior Achilles tendon surgery were excluded from enrollment. All enrolled subjects provided written informed consent, and the study was approved by the Institutional Review Committee of The Third People′s Hospital of Hainan Province.

### Clinical examination

The age, gender, height, weight, body mass index (BMI), fasting glucose levels and blood pressure of all subjects were recorded. Two milliliters of venous blood was collected after overnight fasting and centrifuged within an hour for the measurement of serum LDL-C which was completed using a standard method at the Department of Laboratory Medicine of The Third People′s Hospital of Hainan Province.

**Fig. 1:**
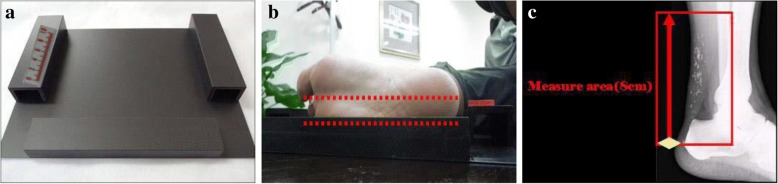
Measurement of ATT by digital radiography. Panel (**a**) shows the measuring platform and ruler. For ATT measurement, the subject was side-lying with the leg and the foot forming a 90 degree angle (panel **b**) and the thickest part of Achilles tendon between the heel and 8 cm above the heel was measured (panel **c**)

ATT was measured by standardized digital radiography with the following imaging parameters: 50 kV, 5 mAs, 0.05 mSv/image, and the distance from the X-ray source to the Achilles tendon was 120 cm [8]. Subjects were positioned with the leg and the foot forming a 90 degree angle. The thickest part of Achilles tendon, between the heel and 8 cm above the heel, was measured (Fig. 1).

### Statistical methods

Statistical analyses were carried out using the SPSS statistical package (19.0). Continuous variables were presented as mean values ± standard deviation (SD), and categorical variables are expressed as percentages and analyzed by the Chi-square test. A one-way analysis of variance followed by the Least Significant Difference (LSD) t test was performed to compare continuous variables. Pearson’s correlation was used to analyze the correlation between the level of LDL-C and ATT. Univariate and multivariable logistic regression models were used to analyze the risk factors of ATT. *P* < 0.05 was considered statistically significant.

## Results

### Study subjects

**Fig. 2:**
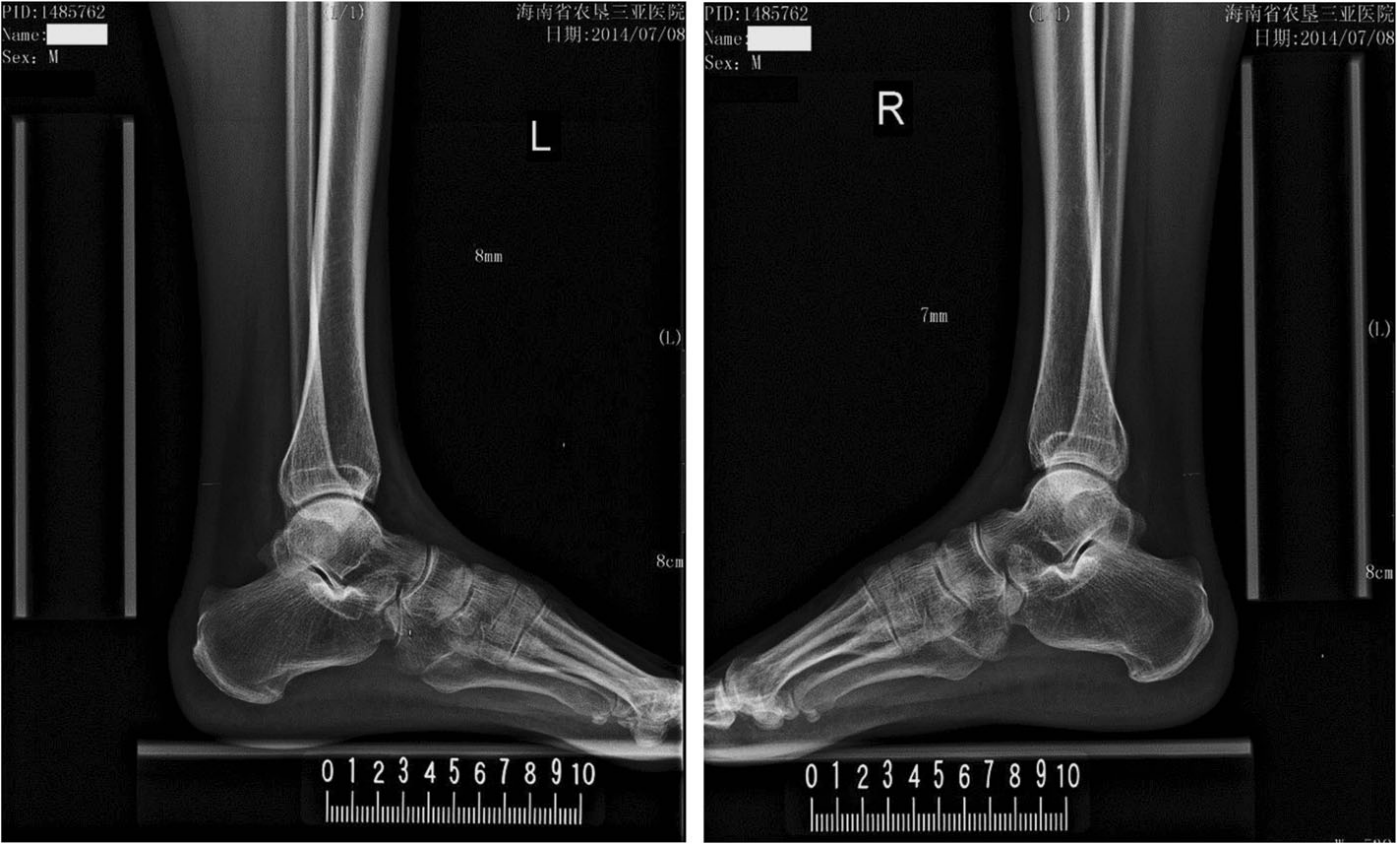
Representative images of ATT measurement in a subject with normal LDL-C. A male who was 66-year-old had an ATT of 7.5 mm (his serum LDL-C was 2.97 mmol/L)

**Fig. 3:**
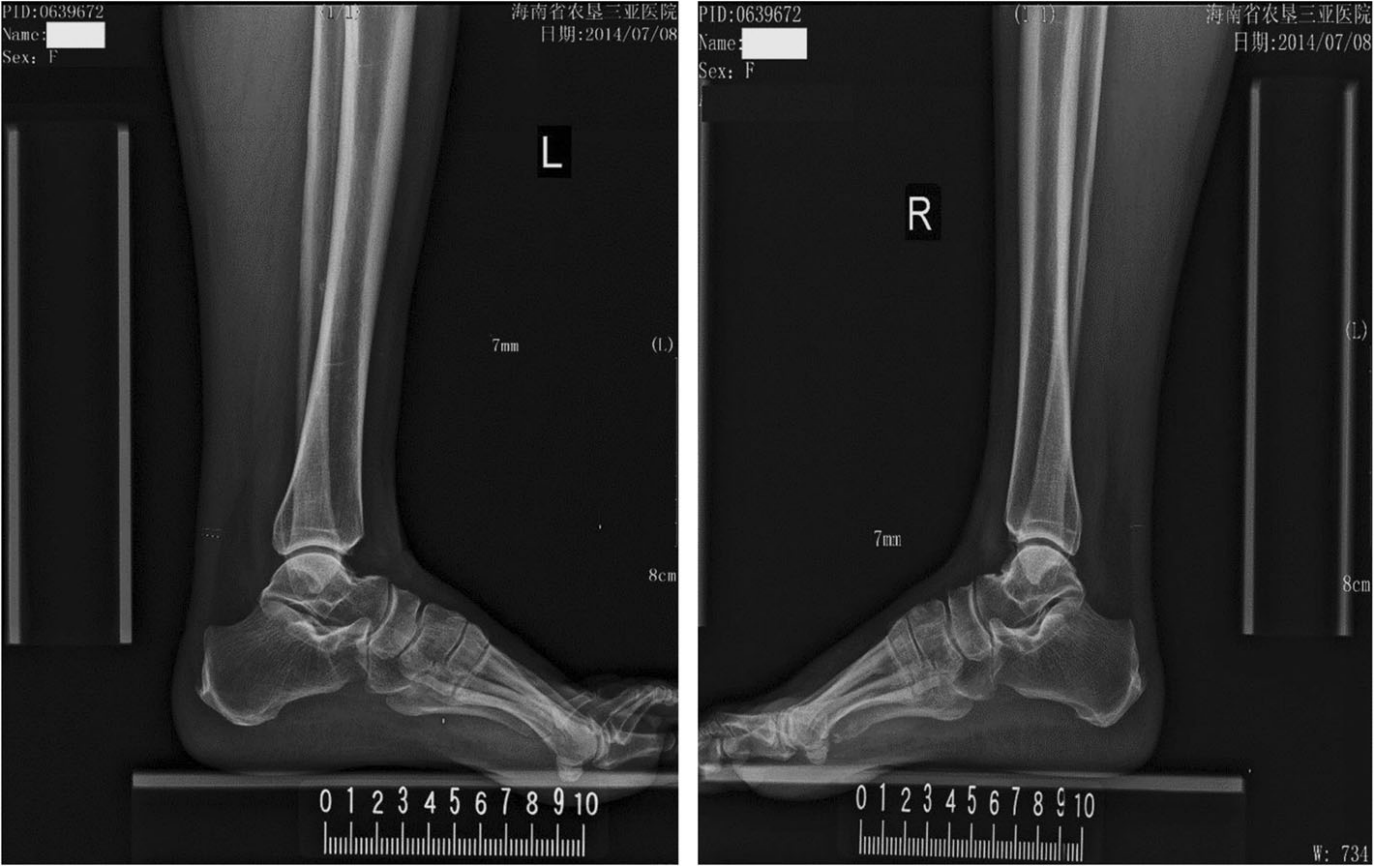
Representative images of ATT measurement in a subject with borderline LDL-C. A female who was 58-year-old had an ATT of 7.0 mm (her serum LDL-C was 3.85 mmol/L)

**Fig. 4:**
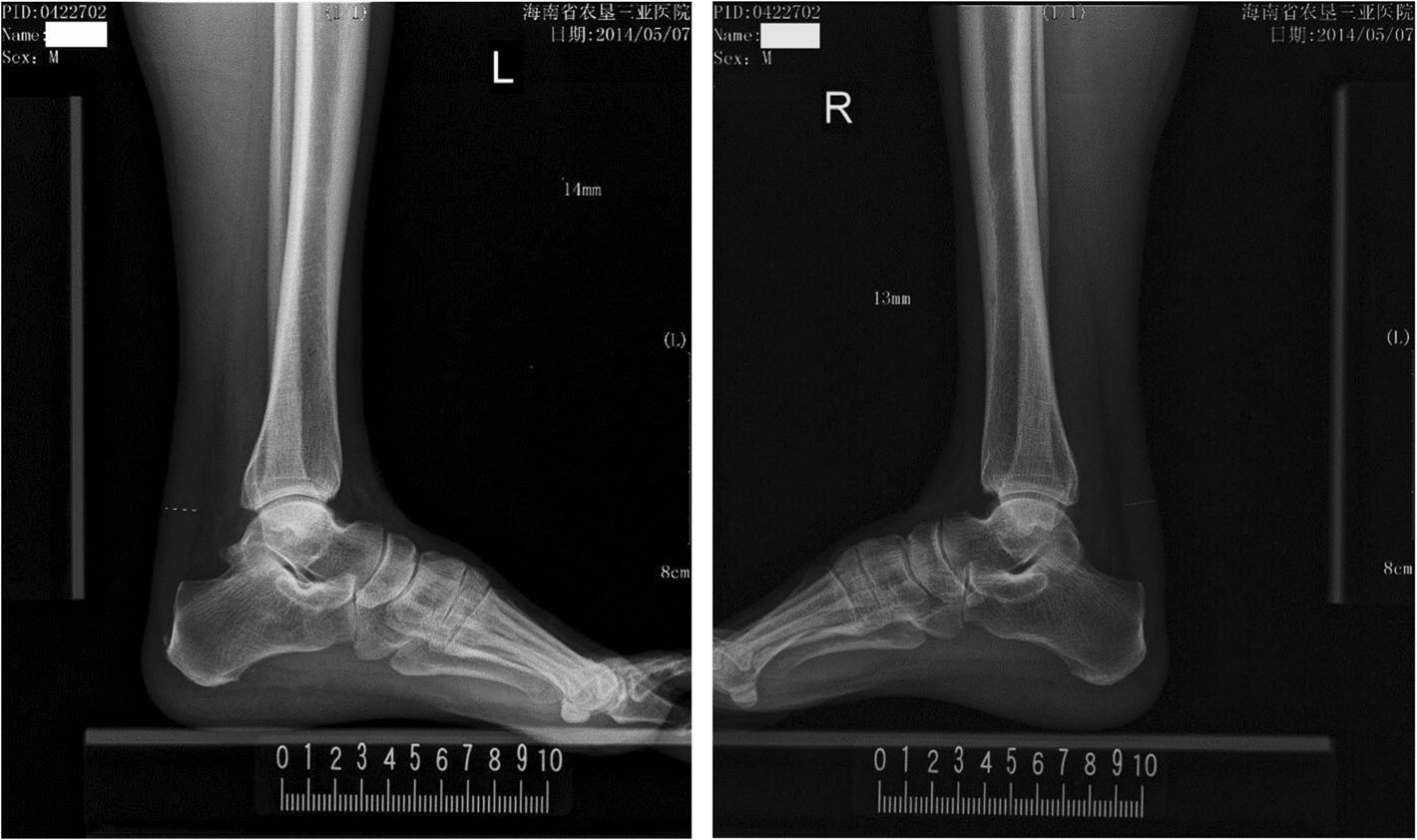
Representative images of ATT measurement in a patient with hypercholesterolemia. A male who was 67-year-old had an ATT of 13.5 mm (his serum LDL-C was 5.46 mmol/L)

The demographic information and clinical characteristics of all 3 groups are shown in Table 1. There were no significant differences in gender ratio, age, height, weight, BMI and blood pressure (systolic and diastolic blood pressure) among the three groups. Four individuals had systolic hypertension (systolic blood pressure average > 140 mmHg on repeated measurements) in the normal LDL-C group, 15 in the borderline LDL-C group and 22 in high LDL-C group. There were 0, 8, and 9 cases of diabetes in normal LDL-C, borderline LDL-C, and high LDL-C groups respectively. Patients in the high LDL-C group had the highest ATT, followed by those in the borderline LDL-C group. ATT was lowest in the normal LDL-C group. Statistical analysis showed that ATT in the borderline LDL-C group was markedly higher than that of the normal group, and ATT in the high LDL-C group was significantly higher than in the other 2 groups (Table 1). Representative images of ATT measurement in individuals from each group are shown in Figs. 2, 3, and 4.

**Table 1:** Demographics and clinical characteristics of all groups

	Normal	Borderline LDL-C	hypercholesterolemia
Number (M/F)	*n* = 51 (26/25)	*n* = 50 (28/22)	*n* = 104 (54/50)
Age (years)	56.90 ± 7.46	51.52 ± 9.57	53.13 ± 10.86
Height (cm)	161.80 ± 6.02	160.61 ± 6.54	161.78 ± 7.27
Weight (kg)	56.55 ± 5.34	59.00 ± 11.28	60.55 ± 10.21
BMI (kg/m^2^)	21.56 ± 1.01	22.75 ± 3.34	23.07 ± 3.00
SBP (mmHg)	139.50 ± 19.78	128.16 ± 12.95	132.65 ± 14.78
DBP (mmHg)	86.00 ± 11.59	80.89 ± 10.34	85.33 ± 10.03
ATT (mm)	6.05 ± 0.28	8.24 ± 1.73*	9.42 ± 3.63^∆#^
Comorbidities			
Hypertension	*n* = 4	*n* = 15	*n* = 22
Diabetes	*n* = 0	*n* = 8	*n* = 9

Continuous data were expressed as mean ± standard deviation

*M* male, *F* female, *BMI* body mass index, *SBP* systolic blood pressure, *DBP* diastolic blood pressure, and *ATT* Achilles tendon thickness

* *P* < 0.05 compared with normal group; ∆ *P* < 0.05 compared with borderline LDL-C group; and # *P* < 0.005 compared with normal group

### Correlation between ATT and LDL-C levels

**Fig. 5:**
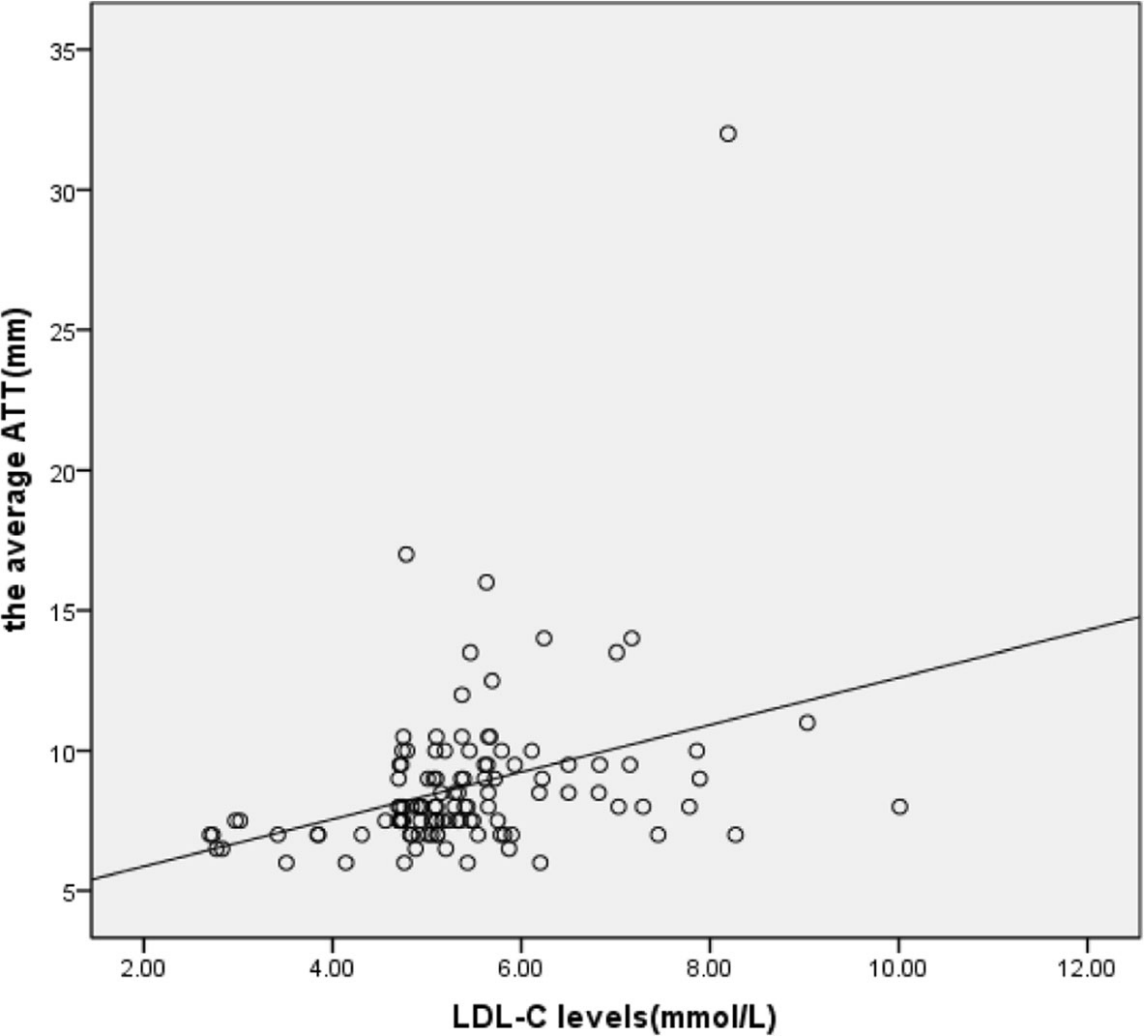
Analysis of correlation between ATT and LDL-C levels. The correlation coefficient was 0.346 between Achilles tendon thickness and serum LDL-C levels (*P* < 0.001)

Analysis of correlation between ATT and LDL-C levels showed a correlation coefficient of 0.346 (*P* < 0.001), and there was a positive correlation as shown in Fig. 5.

### Results of univariate logistic regression analysis

As shown in Table 2, univariate logistic regression analysis using ATT as the dependent variable, and gender, age, height, weight, BMI, systolic blood pressure, diastolic blood pressure, the fasting glucose level, levels of total cholesterol (TC), high density lipoprotein cholesterol (HDL-C), LDL-C, ApoA1, ApoB and lipoprotein(a) (Lp(a)) as independent variables revealed that height, BMI, TC, HDL-C, LDL-C and ApoA1 were associated with ATT (*P* < 0.05).

**Table 2:** The results of univariate logistic regression analysis

Variable	β	S.E	Wald	*P* value	OR	95% CI
Gender	−0.602	0.408	2.183	0.120	0.548	0.246-1.217
Ages	0.010	0.020	0.229	0.632	1.010	0.971-1.050
Height	0.062	0.030	4.160	0.041	1.064	1.002-1.129
Weight	0.006	0.011	0.371	0.542	1.007	0.986-1.028
BMI	0.196	0.068	8.271	0.004	1.271	1.064-1.390
TC	0.277	0.134	4.243	0.039	1.139	1.014-1.715
LDL-C	0.869	0.239	13.274	0.000	2.385	1.494-3.807
HDL-C	−3.573	0.720	24.628	0.000	0.028	0.007-0.115
Apo AI	−5.084	1.027	24.484	0.000	0.006	0.001-0.046
Apo B	−0.209	0.533	0.153	0.695	0.812	0.285-2.238
Lp (a)	0.001	0.001	0.983	0.321	1.001	0.999-1.003
SBP	0.008	0.014	0.295	0.587	1.008	0.980-1.036
DBP	0.005	0.019	0.078	0.779	1.005	0.968-1.044
FGL	0.036	0.071	0.259	0.611	1.037	0.902-1.191

*BMI* body mass index, *TC* total cholesterol, *LDL-C* low density lipoprotein cholesterol, *HDL-C* high density lipoprotein cholesterol, *SBP* systolic blood pressure, *DBP* diastolic blood pressure, *FGL* fasting glucose level

### Results of multivariable regression analysis

Multivariable regression analysis was performed using indexes which were demonstrated to be related to ATT in univariate analysis. The results showed that only the level of LDL-C remained a risk factor for ATT, while levels of HDL-C and ApoA1 were protective factors of ATT (Table 3).

**Table 3:** The results of multivariable logistic regression analysis

Variable	β	S.E	Wald	*P* value	OR	95% CI
Height	0.071	0.047	2.269	0.132	1.073	0.979-1.177
LDL-C	0.626	0.287	4.779	0.029	1.871	1.067-3.280
HDL-C	−2.310	0.894	6.668	0.010	0.099	0.017-0.573
Apo A1	−3.363	1.263	7.085	0.008	0.035	0.003-0.412

*LDL-C* low density lipoprotein cholesterol, *HDL-C* high density lipoprotein cholesterol

## Discussion

Hypercholesterolemia, diagnosed based on blood lipoprotein profiles with TC ≥ 6.22 mmol/L or LDL-C ≥ 4.14 mmol/L [10], can result in deposition and accumulation of cholesterol-rich materials on tendons forming tendon xanthomas. As the Achilles tendon is a common site of lipid deposition, its thickness can provide an early indicator of xanthoma formation [7]. Although ultrasonography has been used for the measurement of ATT [11–15], the methodology has not been standardized [8]. As there is no neighboring tissue to serve as a reliable reference, the subjective nature of ultrasonography for the evaluation of diffuse changes in tendon echogenicity is an additional limitation [10]. Therefore, in this study we adopted a standardized digital radiography method to measure ATT and found that; 1) ATT was significantly higher in patients with hypercholesterolemia than in the other 2 groups, 2) ATT was positively correlated with serum LDL-C levels and, 3) the serum LDL-C level was an independent risk factor, while the HDL-C and ApoA1 levels were protective factors of ATT.

The presence of tendon xanthomas has been recognized as a diagnostic marker for FH [5, 11–17]. However, few studies have explored the association between ATT and LDL-C levels. Ebeling et al. reported that patients with heterozygous familial hypercholesterolemia had higher ATT compared with healthy controls, and ATT was positively related to total cholesterol levels [16]. Junyent et al. observed that, compared with other dyslipidemic or normolipidemic controls, patients with FH had higher ATT which was positively correlated with LDL-C levels [17]. These data align well with our findings, suggesting increased LDL-C levels might be responsible for Achilles tendon thickening. We have also shown that those with borderline LDL-C levels had significantly higher ATT compared with the normal LDL-C group.

It has been shown that ATT is a clinical marker to identify patients at high risk for CVD [9]. We found that the serum LDL-C level was an independent risk factor, while HDL-C and Apo AI levels were protective factors, for ATT. Therefore, LDL-C reduction would be important for the reduction of ATT and the risk of CVD. Indeed, the HMG CoA reductase inhibitor atorvastatin has been shown to reduce ATT in patients with hypercholesterolemia [18] while treatment with simvastatin has been shown to result in regression of Achilles tendon xanthomas [19]. While effective in reducing low-density lipoprotein cholesterol levels and decreasing cardiovascular events, the use of HMG CoA reductase inhibitors can be limited due to patient intolerance from side effects. In that respect, nutraceuticals and functional food ingredients have been recommended as suitable and amiable alternatives for control of dyslipidemia [20]. For those patients with borderline LDL-C and those who simply do not prefer medications, nutraceuticals and functional food ingredients might be the preferred treatment choice.

Limitations of this study include 1) the sample size is small, limiting the generalizability of the results; and 2) the study was carried out in a single center. Multi-centered studies in large series are therefore needed to validate these findings.

## Conclusions

ATT might serve as a valuable auxiliary diagnostic index for hypercholesterolemia and used for the assessment and management of CVD.

## Abbreviations

ATT: Achilles tendon thickness
BMI: Body mass index
CVD: Cardiovascular disease
DBP: Diastolic blood pressure
DR: Digital radiography
FH: Familial hypercholesterolemia
HDL-C: high density lipoprotein cholesterol
LDL-C: low density lipoprotein cholesterol
SBP: Systolic blood pressure
TC: Total cholesterol
